# The impact of coercive and assertive communication styles on children’s perception of chores: an experimental investigation

**DOI:** 10.3389/fpsyg.2024.1266417

**Published:** 2024-02-06

**Authors:** Marius Marici, Remus Runcan, Gheorghe Cheia, Gheorghe David

**Affiliations:** ^1^Education Sciences Faculty, Department of Education Sciences, Ștefan cel Mare University, Suceava, Romania; ^2^Faculty of Educational Sciences, Psychology and Social Work, ‘Aurel Vlaicu’ University of Arad, Arad, Romania; ^3^Faculty of History and Geography, Department of Geography, Ștefan cel Mare University, Suceava, Romania; ^4^Department of Agricultural Technologies, Faculty of Agriculture, University of Life Sciences ‘King Mihai I’ from Timisoara, Timisoara, Romania

**Keywords:** assertive communication, aggressive communication, domestic chores, childrens’ perception, experiment

## Abstract

**Introduction:**

This article presents the findings of an experimental study aimed at investigating the impact of coercive and assertive communication on children’s emotional responses and behavioral tendencies within parent–child interactions.

**Methods:**

The study tested four hypotheses related to children’s feelings, personalization bias, the need to express their point of view, and the desire to retreat to their room alone. Short audio stimuli recorded by a female assistant, representing a mother addressing her child, were utilized to create five different communication situations. The experimental procedure involved participants listening to the audio stimuli and answering related questions. The study included 123 participants between the ages of 9 and 13, with an equal gender distribution.

**Results:**

The results of One-Way ANOVA tests indicated significant differences among the four types of communication in terms of unpleasant feelings, personalization bias, listening to a personal point of view, and retreating into a personal room. The findings suggest that coercive communication elicited more negative emotional responses and stronger tendencies toward personalization bias, expressing personal opinions, and seeking solitude compared to assertive communication.

**Discussion:**

The implications of these findings highlight the importance of promoting positive and respectful communication strategies in parent–child relationships to foster children’s emotional well-being and healthy behavioral development.

## 1 Introduction

Scientific literature has already pointed out, through traditional theories, that coercitive parenting in communication does not support healthy emotional outcomes or prosocial behaviors. Albert Bandura’s Social Learning Theory (Bandura and Walters, 1963) indicates that coercive communication modeled by parents may lead to the internalization of aggressive communication patterns by children. Attachment Theory, developed by Bowlby (1978) shows that harsh or critical communication may undermine the sense of security and trust in the parent–child relationship. Patterson’s Coercion Theory (Patterson, 2016) shows that coercive communication cycles, where negative interactions escalate, may contribute to ongoing behavioral challenges in children. The Transactional Model of Development (Sameroff, 2009) indicates that parents using coercive communication may engage in negative transactions, fostering an unhealthy communication climate. In addition, parents using coercive communication may experience increased conflict with their children, impacting the overall quality of the parent–child relationship (Marici, 2015a). From the perspective of Self-Determination Theory (Deci and Ryan, 2008), coercive communication may hinder the development of a supportive and autonomy-promoting family environment. The Cognitive-Behavioral Model (Carter et al., 2008) suggests that children exposed to coercive communication may develop negative thought patterns and beliefs about themselves and their relationships.

The study is focused on investigating the effect of shouting, negative personality references, ordering, threatening with punishment or being assertive and polite on feeling unpleasant, personalization bias, the need to express one’s personal point of view regarding the issue under discussion or the need to retreat in their room alone. The activity of doing chores was chosen as a pretext, or one action from many other possibilities, in order to investigate the parent–child interactions, as it is very common and parents demand doing chores on a daily basis.

## 2 Raised voice

A raised voice, negative personality reference (“You stupid!,” “You never do your job”), ordering, or threatening are all related concepts that fall into the category of psychological control. There are few studies on the tone of voice of mothers, with this concept generally being incorporated into the concept of “psychological control” or synonymous concepts. A raised voice is a disturbing factor for children, as well as for other categories of people such as partners in a couple, workers, or even in counseling. Studies show that even animals react to harsh tones, and scientists mention “emotional contagion’ as a kind of mirroring of emotions (Maigrot et al., 2022).

The human voice is a critical social cue, and listeners are extremely sensitive to the voices in their environment’ (Abrams et al., 2016, p. 6295). Even from a young age, infants exhibit nonnutritive sucking behavior when hearing their mother’s voice with normal intonation, compared to when she speaks without the prosodic and intonation aspects of normal speech (Mehler et al., 1978). At infancy, the mother’s voice serves as a non-noxious intervention with positive effects on care and child development (Provenzi et al., 2018). As children reach around the age of 13, their brains shift focus from familiar voices, like their mothers’, to new and unfamiliar ones, preparing them for eventual separation from their parents (Abrams et al., 2016). An autonomy-supportive and motivating tone of voice in adolescents is positively related to emotions, closeness, and intentional behavioral engagement, whereas a controlling tone is associated with the opposite reactions (Weinstein et al., 2019).

A controlling tone is associated with less positive personal and interpersonal outcomes, such as perceived choice, lack of perceived pressure, well-being, closeness to people, and prosocial behaviors. An autonomy-supportive tone serves as a motivating factor in communication (Weinstein et al., 2018). The use of harsh verbal discipline (including tone of voice, but not limited to it) by both mothers and fathers toward their 13-year-old children has been found to be a significant predictor of an escalation in adolescent conduct problems and depressive symptoms between the ages of 13 and 14 (Wang and Kenny, 2014).

## 3 Negative personality references

Negative personality references, as defined by parental statements attributing negative or positive personality traits to children based on their actions, can have detrimental effects on children’s psychological well-being. These references are influenced by the social norms of various social groups, and disobedience of these norms can result in punishments. Labeling theory posits that individuals are labeled as deviant or wrong when their behavior is recognized as such by others (Hagan, 1973). However, Becker (1963) suggests that an increase in labeling leads to an increase in deviance among children. Negative personality references might occur in the contexts of parental aggressions, bullying or verbal abuse.

According to the study conducted by Johnson et al., 2001, there was compelling evidence indicating that individuals who encountered maternal verbal aggression in their childhood had a more than threefold increased likelihood of developing borderline, narcissistic, obsessive-compulsive, and paranoid personality disorders during their adolescence or early adulthood. According to Zhang’s research in Zhang (1997), the way young individuals perceive the labels assigned to them by their parents can generate emotions of social rejection or isolation. The youth tend to feel isolated from the individuals who assigned the labels. In other words, if parents label them, they experience a sense of isolation from their parents.

The labeling theory posits that the labels assigned to children influence their self-perception and behavior as they internalize and conform to the associated expectations (Becker, 1963). This process aligns closely with the concept of self-fulfilling prophecy, where the labels assigned to children can manifest as reality as their behavior aligns with the expectations associated with the labels (Merton, 1948). Moreover, the theory of stereotype threat highlights how negative stereotypes linked to a child’s social group can impede their performance and behavior due to the fear of confirming those stereotypes (Steele and Aronson, 1995). These processes are influenced by social identity theory, which asserts that children’s self-concept and behavior are shaped by the labels associated with their social groups, thereby impacting their interactions and sense of identity (Tajfel et al., 1979). Finally, symbolic interactionism emphasizes that labels assigned to children are socially constructed through interactions, shaping their self-concept and subsequent interactions with others (Blumer, 1986). Collectively, these theoretical perspectives provide valuable insights into how labeling influences children’s experiences, self-perception, and behavior within social contexts.

## 4 Ordering children to comply

Authoritarian parenting styles play a crucial role in shaping a child’s psychological development. While setting expectations and fostering discipline are essential aspects of child rearing, demanding harshly from children can have adverse effects on their psychological dynamics (Trifan et al., 2014).

Demanding harshly from children can also lead to a reduction in positive emotions (Hecker et al., 2016). When children constantly face criticism and pressure, their ability to experience joy, happiness, and positive emotions may be hindered. This can result in a diminished sense of overall well-being and a negative emotional state.

Demanding harshly from children can contribute to negative self-perception and a distorted self-image. Children may internalize the belief that they are inherently flawed or inadequate, leading to negative self-talk and low self-esteem. These negative self-perceptions can reinforce personalization bias, as children are more likely to attribute negative outcomes solely to themselves rather than considering external factors (Marici, 2015a). This self-blame can contribute to personalization bias, where children excessively attribute negative events to themselves (Marici et al., 2023).

Demanding harshly from children can have detrimental effects on their need to be listened to, as children suppress their emotions (Cameron and Overall, 2018). It suppresses their expression and inhibits their ability to assert their needs and preferences. Children may feel a diminished sense of agency and perceive themselves as passive recipients of directives. Communication barriers may arise, hindering their ability to openly communicate and share their experiences. Trust and emotional safety within the parent–child relationship can be eroded, causing children to hesitate in expressing themselves due to fear of negative reactions.

Harsh demands directed at children can foster the development of avoidance or withdrawal behaviors, as the children may develop fear toward their parents (Pérez et al., 2021). Children may withdraw socially, avoiding interactions and situations where they fear judgment or criticism. The fear of failure and the erosion of self-confidence can further contribute to retreating. Harsh parenting practices hinder the development of social skills, limiting children’s ability to initiate conversations and form connections. The emotional consequences include increased anxiety, shame, and low self-esteem (Marici et al., 2023). It is crucial for parents and caregivers to recognize the impact of their actions and create a supportive environment. Constructive feedback, nurturing, and emotional safety can help mitigate retreating behaviors and encourage children to confidently engage in social interactions and personal growth.

## 5 Threatening with punishment

The effect of threatening children with punishment, a disciplinary strategy often employed by parents and caregivers, has been a subject of investigation in psychological research. Threats of punishment can have several effects on children’s behavior and well-being.

Threatening children with punishment can elicit fear and anxiety, as it creates an aversive and unpredictable environment. This can lead to negative emotional and psychological consequences, such as increased stress levels, reduced self-esteem, and a strained parent–child relationship (Gershoff, 2013). The fear induced by punishment threats may also hinder children’s cognitive functioning and impair their ability to learn and problem-solve effectively (Skinner and Belmont, 1993). Moreover, the use of punishment threats can shape children’s behavior through external compliance rather than internalization of values and morals (Marici, 2015a,b). Children may comply with the desired behavior to avoid punishment rather than understanding the reasons behind the rules. This can inhibit the development of self-regulation skills and the internalization of appropriate social norms (Grusec and Goodnow, 1994). Additionally, threatening children with punishment may foster a punitive and hostile family climate, where negative interactions and power dynamics prevail. This can negatively impact the parent–child relationship, leading to reduced trust, communication difficulties, and an erosion of emotional connection (Larzelere et al., 2004; Turliuc and Marici, 2013a). Children may become more resistant, defiant, or withdrawn in response to frequent punishment threats.

When children are consistently threatened with punishment, the experience of fear and anxiety may lead them to seek a place of safety or solitude, often their own room (Hudson and Rapee, 2001). Threatening children with punishment can lead to their withdrawal behaviors, such as retreating to their room (Masten and Cicchetti, 2010). This response is driven by the fear and anxiety induced by consistent punishment threats (Crick and Dodge, 1994). Children may seek their room as a place of safety and solitude, distancing themselves from the perceived threat. Withdrawing to their room can serve as a protective mechanism and a form of avoidance to avoid further conflict. While it may provide temporary relief and a chance to regulate emotions, excessive withdrawal can hinder social development.

When children are repeatedly threatened with punishment, they may begin to perceive themselves as inherently flawed or responsible for negative events (Dunsmore and Halberstadt, 1997). This bias can lead to negative self-perceptions, low self-esteem, and a heightened sense of guilt or shame. The fear and anxiety induced by punishment threats can amplify this personalization bias, reinforcing the belief that they are the cause of negative outcomes. Threatening children with punishment has been found to be linked to the occurrence of the personalization bias, whereby individuals interpret events as specifically targeted toward them (Eberly and Montemayor, 1998). This cognitive bias can lead children to perceive ambiguous situations as personally directed, even when they are not (MacEvoy et al., 1999). The personalization bias can negatively affect children’s emotional well-being and social interactions (Leary et al., 2006). Harsher forms of punishment have been associated with a stronger personalization bias, indicating a dose–response relationship (Gershoff et al., 2010). However, further research is needed to establish causality and understand the underlying mechanisms. Alternative disciplinary strategies that emphasize positive reinforcement and open communication are crucial for mitigating the potential negative impact of the personalization bias on children (Sanders et al., 2004).

Threatening children with punishment may potentially affect their perception of being listened to in regard to their personal points of view. When children are consistently exposed to threatening language or punishment, it may create an environment that discourages open communication and inhibits their willingness to express their thoughts and perspectives (Kochanska et al., 2003). This could lead to a diminished sense of being heard or valued, as their personal points of view may be disregarded or dismissed (Grusec and Goodnow, 1994).

## 6 Assertive communication

Assertive communication is a form of interpersonal interaction characterized by the expression of one’s thoughts, feelings, and needs in a direct and respectful manner. It encourages participation, involvement and staying in the conversation. It involves clear and concise statements that convey confidence and self-assurance, while also acknowledging and respecting the perspectives of others (Ward and Holland, 1997). This approach fosters effective and open communication, enhancing mutual understanding, and promoting positive outcomes in social and professional contexts (Alberti and Emmons, 2017). Assertive communication promotes wellbeing as opposed to all forms of aggressive communication, discourages personalization biases, and social interactions. Scientific literature has already documented that assertive communication leads to better psycho-social outcomes in human interactions.

## 7 Research methodology

### 7.1 The present study

The aim of this study is to investigate the effects of coercive and assertive statements on children’s emotional responses and behavioral outcomes. Using vignettes as the foundation, the study examines how different communication styles used by mothers in parent–child interactions impact children’s reactions to specific situations.

### 7.2 Hypotheses

For the present study we had four hypotheses:

Hypothesis 1: Children exposed to coercive statements will exhibit heightened levels of unpleasant feelings in response to their mother’s statements, compared to when they are exposed to an assertive statement.

Hypothesis 2: Coercive statements are predicted to elicit a greater frequency of the personalization bias, in contrast to the assertive statement.

Hypothesis 3: Children who listened to coercive statements are expected to demonstrate a stronger proclivity to assert their point of view regarding the discussed issue, in comparison to children who listened to the assertive statement.

Hypothesis 4: Children exposed to coercive statements are anticipated to express an increased desire to seek solitude in their room, as opposed to children exposed to assertive statements.

### 7.3 Instruments

The present study had two components regarding the instruments used: it contained several questions and vignettes, all developed by the research team.

#### 7.3.1 The questions used

We used four question-type items:

a) This item measures “unpleasant feelings”: “If your mom speaks to you like this, how much does it bother you?’ (Not at all 0 1 2 3 4 5 6 7 8 9 Very much),
b) This item measures “personalization bias”: “How much do you think your mom has something personal against you because she addresses you this way?’ (Not at all 0 1 2 3 4 5 6 7 8 9 Very much),
c) This item measures “the need for one’s personal point of view to be listened”: “How much do you want your mom to listen to your point of view after she says this?’ (Not at all 0 1 2 3 4 5 6 7 8 9 Very much),
d) This item measures “the need to retreat into one’s own room”: “How much do you want to be left alone and retreat to your room by yourself?’ (Not at all 0 1 2 3 4 5 6 7 8 9 Very much).
e) In addition, demographic data was collected about participants such as: age, sex, or parents’ income. Although parents’ income data is harder to collect, we asked children to indicate their perception on the fact whether parents earn much more than needed, more than needed, exactly as needed, less than needed or very little or at all. This way children could approximate their parents income.

#### 7.3.2 Experimental procedure

The children were told that they are going to hear a description of a life situation (vignettes) and listen to a short audio recording, uttered by a mother, who solicits her child to do something. There will be 5 such situations, which will follow one after another (phase 4).

#### 7.3.3 Phases of the procedure of vignettes development

**Figure 1:**
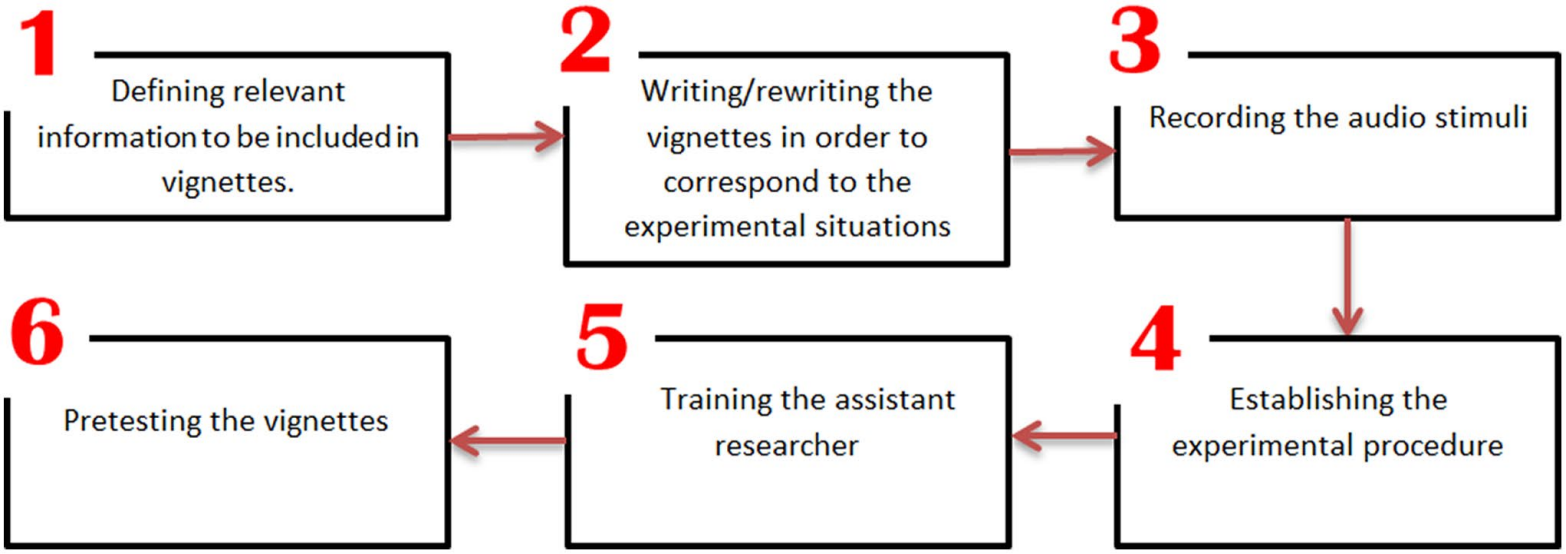
The measurement instrument development flowchart.

To examine the formulated hypotheses, we devised an assessment tool employing vignettes as the foundation. Figure 1 illustrates the successive stages we traversed during the development process of the ultimate measurement instrument.

#### 7.3.4 The process of vignette creation

The vignettes were created by a group of experts during the *first* phase. These experts selected five scenarios, determining what each scenario should include (proximal) and exclude (distal). Four instances of coercive communication were selected due to their frequency in parent–child interactions, while assertive communication was chosen as it represents the optimal mode of communication.

The current research integrates both acoustical characteristics and the content of communication in stimuli, encompassing five distinct communication scenarios. This implies that the stimuli (vignettes) vary in terms of acoustics and content. It is important to note, however, that the experiment did not aim to manipulate specific sound characteristics in the audio, such as pitch, amplitude, or duration. Instead, the focus was on the overall semantic aspects of the vignettes. These vignettes were developed by incorporating qualitative, semantic characteristics and were subsequently semantically tested by respondents of the same age category to ensure they met the criteria required for the experiment. Considering the semantic aspect of the vignettes, the study can be replicated.

In the *second* phase, all five communication instances involved the child being asked to tidy the personal room. The vignettes asked the children to imagine a scenario involving a mother and her child, where the child’s room is in disarray. Upon seeing this, the mother requests the child to tidy the personal room and says the following. The children then listened to the recorded instances one by one. For all five instances, the children had to imagine the same scenario involving a mother and her child. The experiment clarified that the instances were about a hypothetical child and their mother, not about the participating children and their own parents.

Instance one exemplified a raised voice, with the text being: “I told you one thousand times to tidy up your room.” The tone was high and rapid. Instance two exemplified a negative personality reference, with the text: “You are messy, and you never tidy up your room.” The tone contained elements of mockery and condescension. Instance three represented an order, with the text: “Go now and tidy up your room! Do you understand?” The phrase was spoken forcefully and assertively. Instance four demonstrated a request accompanied by a threat of punishment, with the text: “If you do not tidy up your room now, you are grounded!” The tone was serious and firm. Finally, instance five depicted an assertive situation, with the text: “Please go and tidy up your room, and then come and eat!” The tone was pleasant, featuring a polite voice and a regular rhythm.

#### 7.3.5 Audio stimuli

They were very short audio recordings (phase 3) in the studio, using professional equipment. Thus, they were clear and accurate. We established 5 audio situations that were relevant for our research. All recordings were sentences uttered by a female assistant, as representing a mother addressing her child.

#### 7.3.6 The content of the audio stimuli

The recordings were meticulously crafted to represent five unique situations: (1) the mother addressing her child with a raised, yet non-aggressive voice, (2) the mother making a negative personality reference (using phrases such as “you never”) in a heightened tone, (3) the mother giving an order to her child in a loud tone, (4) the mother initially soliciting her child and then threatening them with punishment for non-compliance, and finally (5) the mother employing an assertive communication style, marked by a pleasant and polite tone. Notably, the first four scenarios fall under the umbrella of coercive parental solicitation, while the last one belongs to the category of assertive and polite communication. These specific coercive communication styles were selected for their prevalence in parent–child interactions and their classification as psychological control practices. Despite a majority of research focusing on the exploration of coercive communication through multi-item scales, few studies have investigated the impacts of individual practices, often measured through a single item.

#### 7.3.7 Assistant training

Nevertheless, prior to the commencement of the experiment, a training exercise was conducted with the assistant researchers (phase 5), during which they were guided through all the steps of the experiment. Any questions were addressed and further instructions were provided to ensure a clear understanding of the experimental procedure. Subsequently, these assistant researchers were put through a practical test in real-life situations, wherein they were tasked with administering the experiment to a select group of individuals. This served as a preparatory measure for the actual experiment. After this stage, a follow-up session was conducted where further instructions were given to guarantee a seamless execution of the real experiment.

#### 7.3.8 Vignette testing

After the vignettes were recorded we tested (phase 6) them on a number of participants.

The participants in the pretest were distinct from those involved in the main experiment, although they belonged to the same age group as the participants in the actual experiment. They were children between 9 and 13 years old, from Suceava, Romania, and their distribution according to gender was almost equal and nonsignificant. The pretest respondents were tested quantitatively using a questionnaire. The participants were informed that they are going to listen to several stimuli statements uttered by a mother to her child, asking the child to comply and do household chores. They have to listen to the statements and answer several questions about the statements. The assistant researcher made the introduction presenting the experiment, and the respondents were handled several sheets of paper with the questions to fill in. We used a personal computer with stereo speakers attached, to play all audio stimuli. The design was a within-subjects design and all participants evaluated all five audios. The audios were played one by one in this order: “screams at me,” “blames me,” “forces me,” “punishes me,” and “speaks nicely with me.” The respondents were given enough time to fill in their answers between auditions. If they needed help they were assisted by the assistant researcher. The problems that arose were about not understanding the question, requesting confirmations about how to fill in, or where to write.

For the pretest section we had two objectives. Firstly, we wanted to test whether the respondents can identify each statement as belonging to its category, and secondly, we intended to apply the whole experiment procedure to test it and identify possible problems. For the first objective we asked participants to listen to each audio stimulus in part and attribute it to the right category. The categories were: “speaks nicely with me,” “punishes me,” “screams at me,” “blames me,” “forces me.” The SPSS results indicated that all audio stimuli were attributed to the right category, with the highest frequency (see Table 1).

**Table 1:** The results of pretesting vignettes, specifically audio files, and their corresponding categories.

Audio files and categories	*N*	*%*	Chi^2^ test
*1. Audio “Screams at me”*			
punishes me	4	3.7	
blames me	6	5.6	
speaks nicely with me	10	9.3	
forces me	32	29.6	Chi^2^(1) = 4.067, *p* = 0.0437, Cramér’s *V* = 0.194^1^
screams at me	56	51.9	
*2. Audio “Blames me”*			
punishes me	6	5.6	
forces me	8	7.4	
screams at me	11	10.2	
speaks nicely with me	13	12	Chi^2^(1) = 12.267, *p* = 0.0005, Cramér’s *V* = 0.34^2^
blames me	68	64.9	
*3. Audio “Forces me”*			
punishes me	4	3.7	
blames me	6	5.6	
speaks nicely with me	12	11.1	
screams at me	24	22.2	Chi^2^(1) = 8.496, *p* = 0.0036, Cramér’s *V* = 0.28^1^
forces me	62	57.4	
*4. Audio “Punishes me”*			
screams at me	2	1.9	
blames me	6	5.6	
speaks nicely with me	6	5.6	
forces me	16	14.8	Chi^2^(1) = 18.451, *p* = 0.0001, Cramér’s *V* = 0.413^2^
punishes me	78	72.2	
*5. Audio “Speaks nicely” with me*			
punishes me	0	0	
blames me	0	0	
screams at me	2	1.9	
forces me	4	3.7	Chi^2^(1) = 37.926, *p* = 0.0001, Cramér’s *V* = 0.598^3^
speaks nicely with me	100	94.4	

The table provides the frequency (*N*) and percentage (%) for each category, along with the chi-square test statistics and *p*-values.

^1^ = small association, ^2^ = medium association, ^3^ = strong association.

The pretesting indicated that in case of the audio “Screams at me” the respondents attributed this audio the most frequently to “Screams at me” with 51.9%, followed by “Forces me” with 29.6%. The same happens in case of the audio “Forces me” where respondents attributed it the most frequently to “Forces me” (57.4%) followed by “Screams at me” (22.2%) The fact that these percentages are both high might be that they somehow correlate. Screaming at a person transmits the idea of pressuring the person, while pressuring the person requires a dose of screaming at a person. In order to find out whether there are significant differences between the responses tested we performed a Chi squared test (N-1’ Chi-squared test). In order to do that, we ordered ascendingly the scores obtained at each category and performed the Chi squared test between the targeted scores and the next lower score. All tests indicated that the targeted scores are significantly different from the next lower scores. This means that each vignette succeeded in measuring mainly the intended phenomenon (see Table 1).

### 7.4 Experiment participants

The participants were 123 respondents (*M*_age_ = 11.2, SD = 0.699). About 60.2% of them were males and 39.8% were females. Children participating in the experiment aged between 9 and 13 years. The participants were from Suceava city, Romania. About 6.7% of the children had parents who earned much more than they needed, 31.7% had parents who earned more than they needed, 59.2% had parents who earned exactly as much as they needed, and 2.5% had parents who earned less than they needed. Our study had 5 experimental situations and all participants were exposed to all of them in similar proportions. The experiment developed in group setting, in school classrooms.

### 7.5 Ethical considerations

The present research followed the normative ethical considerations in the field in case of minor children. All children received an informed consent and their parents consented that their children participated in the research and sent consent papers signed. About 91% of all participants targeted participated in the experiment. All the participants had the right to withdraw from the research at any moment, and all data collected remained confidential. After they finished filling in the questionnaires, we had a debriefing about the study. We discussed the purpose of the study, how the participants felt, and we gave them feedback about all the questions they had. In the debriefing part the participants indicated by verbal and nonverbal means that they were positive about the experience of participating in the experiment. The researchers communicated to them that they were grateful that they took part in the research. The children were not rewarded for taking part in the experiment.

## 8 Statistical procedure

In order to analyze the data we used IBM SPSS 26 software and One-Way ANOVA method.

## 9 Results

Firstly, we presented the descriptive data. Table 2 presents descriptive statistics for different variables measured in the study, categorized by the type of communication used.

**Table 2:** Descriptive statistics.

Variable measured	Type of communication	*N*	Mean	SD	SE	No.^1^
Unpleasant feelings	Raised voice	123	4.163	2.44	0.220	3.7
	Negative personality references	121	4.446	2.74	0.249	4
	Ordering	123	4.699	2.63	0.237	4.2
	Threatening with punishment	122	5.377	2.99	0.271	4.8
	Assertive	*121*	*1.116*	*2.18*	*0.198*	–
	Overall	610	3.965	2.99	0.121	3.6
Personalization bias	Raised voice	121	2.264	2.53	0.230	2.7
	Negative personality references	121	2.256	2.52	0.229	2.7
	Ordering	123	2.382	2.67	0.241	2.9
	Threatening with punishment	121	3.041	2.98	0.271	3.6
	Assertive	*121*	*0.835*	*1.85*	*0.168*	–
	Overall	607	2.156	2.63	0.106	2.6
Listening to your point of view	Raised voice	123	6.211	2.68	0.242	2.7
	Negative personality references	121	5.579	2.95	0.268	2.5
	Ordering	122	5.549	3.07	0.278	2.4
	Threatening with punishment	124	5.750	3.06	0.275	2.5
	Assertive	*121*	*2.273*	*2.87*	*0.261*	–
	Overall	611	5.080	3.24	0.131	2.2
Retreat into your room	Raised voice	123	4.870	3.34	0.301	2.8
	Negative personality references	122	4.434	3.02	0.273	2.5
	Ordering	122	4.779	3.17	0.287	2.7
	Threatening with punishment	123	4.870	3.39	0.306	2.8
	Assertive	*122*	*1.754*	*2.82*	*0.256*	–
	Overall	612	4.143	3.36	0.136	2.4

^1^Number of times Assertive communication is smaller than any other type of communication.

In order to test our hypotheses we used One-Way ANOVA method in IBM SPSS. The Test of Homogeneity of Variances indicated that the condition of equal variances was not assumed in the following situations: “Unpleasant Feelings condition” [*F*(4, 605) = 9.404, *p* = 0.000)], “Personalization Bias” [*F*(4, 602) = 14.722, *p* = 0.000)], or “Retreat into Personal Room” [*F*(4, 607) = 6.633, *p* = 0.000)], which recommended the Games-Howell test. For the condition “Listening to Personal Point of View” the condition of equal variances was assumed: [*F*(4, 606) = 2.220, *p* = 0.065)].

Table 3 presents the results of a One-Way ANOVA (*Post Hoc*, Games-Howell and Tukey) analysis conducted on four variables.

**Table 3:** One-Way ANOVA.

Variable	*F*	*df1*	*df2*	*p*	*η^2^*
Unpleasant feelings^3^	48.6	4	605	< .001^4^	0.243^1^
Personalization bias^3^	12.2	4	602	< 0.001	0.075^2^
Listening to personal point of view^4^	35.5	4	606	< 0.001	0.190^1^
Retreat into personal room^3^	22.25	4	607	< 0.001	0.127^1^

(1)^1^ = large effect, ^2^ = moderate effect (Cohen, 1988). (2)^3^ Games-Howell *Post Hoc* test was used. (3)^4^ Tukey *Post Hoc* test was used. (4) ^4^Even with a Bonferroni correction applied to all comparisons the p value remains significant. A Bonferroni Correction involves adjusting the alpha (α) level for a set of statistical tests to control for the probability of making a type I error. *p* = 5*0.001 = 0.005; *p* < 0.05 in all situations tested above (Armstrong, 2014).

These results suggest that there are statistically significant variations among the groups in terms of unpleasant feelings, personalization bias, listening to personal point of view, and the tendency to retreat into a personal room. The *p*-values indicate that the observed differences are unlikely to have occurred by chance and highlight the importance of further examining the factors contributing to these variations.

Table 4 presents One-Way ANOVA significant differences observed between groups.

**Table 4:** Significant differences between the groups compared.

Significant differences		Mean difference	*t*	*df*	*p*-value
*Unpleasant condition^1^*					
Raised voice	Assertive	3.05	10.3	120	<0.001
Negative personality reference	Assertive	3.33	10.5	115	<0.001
Ordering	Assertive	3.58	11.6	118	<0.001
Threatening with punishment	Assertive	4.26	12.7	111	<0.001
*Personalization bias^1^*					
Raised voice	Assertive	1.43	5.02	110	<0.001
Negative personality reference	Assertive	1.42	5.01	110	<0.001
Ordering	Assertive	1.55	5.27	109	<0.001
Threatening with punishment	Assertive	2.21	6.92	100	<0.001
*Need for one’s personal point of view to be listened to^2^*					
Raised voice	Assertive	3.94	11.06	120	<0.001
Negative personality reference	Assertive	3.31	8.83	120	<0.001
Ordering	Assertive	3.28	8.59	120	<0.001
Threatening with punishment	Assertive	3.48	9.17	122	<0.001
*Need to retreat into the room alone^1^*					
Raised voice	Assertive	3.12	7.89	119	<0.001
Negative personality reference	Assertive	2.68	7.16	121	<0.001
Ordering	Assertive	3.02	7.87	120	<0.001
Threatening with punishment	Assertive	3.12	7.82	118	<0.001

^1^ = it was used Games-Howell *Post Hoc* Test for unequal variances, ^2^ = it was used Tukey *Post Hoc* Test for equal variances.

The results indicated that there are significant differences between all groups analyzed, between assertive communication condition and the other conditions, with a *p* value significant at lower than 0.001 level.

## 10 Discussion

The present study aimed to investigate the effects of different types of parental communication on children’s responses. The hypotheses proposed in this research were based on the assumption that coercive statements would elicit more negative emotional reactions and maladaptive behavioral tendencies compared to assertive statements. The results of the study supported the hypotheses, as indicated by the significant differences observed among the groups in terms of unpleasant feelings, personalization bias, listening to one’s personal point of view, and retreating into a personal room.

In line with the first hypothesis, children who listened to coercive statements reported more unpleasant perceptions toward their mother’s statements compared to when they listened to an assertive statement. This suggests that coercive communication styles, characterized by raised voices, negative personality references, orders, and threats of punishment, have a detrimental effect on children’s emotional experiences, potentially leading to increased discomfort and distress (Weinstein et al., 2018).

Furthermore, the second hypothesis was supported, indicating that coercive statements activated the personalization bias more frequently than the assertive statement. This result has support in science (Eberly and Montemayor, 1998). Children tend to attribute negative events or criticism to one’s own personal characteristics, in these conditions. This finding suggests that coercive communication may contribute to the internalization of negative attributions and self-perceptions in children, which can have long-term implications for their self-esteem and well-being.

Consistent with the third hypothesis, children who listened to coercive statements reported a higher need to express their point of view regarding the issue under discussion compared to children who listened to the assertive statement. This finding implies that coercive communication may evoke a stronger desire in children to assert their autonomy and have their opinions heard, potentially reflecting their need for agency and autonomy in interpersonal interactions (Marici, 2015a).

Lastly, the fourth hypothesis was supported, indicating that children who listened to coercive statements reported a higher need to retreat into their room alone compared to children who listened to assertive statements. This finding suggests that coercive communication may create an aversive environment for children, leading them to seek solitude and withdrawal as a coping mechanism to deal with the negative emotional and behavioral effects of coercive interactions (Turliuc and Marici, 2013b).

The results of this study highlight the importance of parental communication styles in shaping children’s emotional experiences and behavioral tendencies. The findings support previous research on the detrimental effects of coercive communication practices and provide further insight into the specific mechanisms through which coercive statements can influence children’s emotional and behavioral responses (Marici and Turliuc, 2011; Turliuc and Marici, 2013b).

## 11 Conclusion

Specifically, the study aimed to examine the impact of coercive statements compared to assertive statements, providing empirical evidence for their influence on children’s emotional experiences and behavioral outcomes. The study reiterates the importance of approaching assertive communication in order to avoid unpleasant feelings but it adds further value to research by showing that the personalization bias is growing, and children feel not a weaker but a stronger need to be listened to and to retreat into their room instead of getting involved.

It is worth noting that one of the strong points of the present study is the use of short audio stimuli to simulate real-life parental communication situations. The utilization of professional equipment ensured the clarity and accuracy of the audio recordings, enhancing the ecological validity of the experimental design. The selection of specific audio situations representing coercive and assertive communication categories allowed for a focused investigation of these communication styles and their effects on children’s responses.

However, it is important to acknowledge some limitations of the study as well. The participants were limited to a specific age range (9–13 years) and were drawn from a single city in Romania, which may restrict the generalizability of the findings to other populations. Additionally, the study employed a group setting in school classrooms for data collection, which might have influenced participants’ responses due to social dynamics and peer presence. Moreover, the research presents a predominantly normative outlook on parent–child communication, neglecting cross-cultural investigations that highlight the adaptability of varied communication styles in child-rearing practices globally (Margalit and Mauger, 1985; Omura et al., 2018).

Future research should include a broader age range and more diverse samples to enhance the external validity of the findings. Future studies should also test some other relevant tendencies and behaviors as a response to the stimuli presented in the actual experiment. Conducting the experiment in a controlled individual setting or real situation could provide a more accurate reflection of children’s genuine emotional and behavioral reactions.

In conclusion, this experimental investigation demonstrated that coercive parental communication styles, characterized by raised voices, negative personality references, orders, and threats, have significant effects on children’s emotional experiences and behavioral tendencies. The findings emphasize the importance of promoting assertive and positive communication practices within parent–child relationships, as they are associated with more positive emotional responses and adaptive behaviors in children.

## 12 Implications

These findings offer several practical implications for parents, educators, and mental health professionals.

*Firstly*, the data suggests that educational actors should know how to use assertive communication and avoid coercive communication. Coercive language implies threats, orders, and harsh tones which can lead to negative consequences in children. Parents should express their needs and instructions in a respectful manner, using clear, direct language and avoiding raised voices and threats. *Secondly*, given the link between coercive communication and the activation of personalization bias, parents need to be aware that their children might internalize negative criticism as reflective of their character. *Thirdly*, rather than suppressing children’s’ desire for autonomy, parents should respect and nurture it, promoting open dialog where the child’s opinion is valued. *Lastly*, the tendency for children to retreat into solitude as a response to coercive statements underscores the need for educational actors to create a nurturing and comfortable environment. If a child regularly seeks solitude following parental interactions, this could signal that the child perceives the environment as aversive. Mutual respect and understanding, feeling safe and comfortable and expressing their feelings are a necessity for children.

These results reinforce the significance of using non-coercive, assertive communication when interacting with children.

## Data availability statement

The raw data supporting the conclusions of this article will be made available by the authors, without undue reservation.

## Ethics statement

The studies involving humans were approved by Aurel Vlaicu University of Arad, Faculty of Educational Sciences, Psychology and Social Work, Center of Research Development and Innovation in Psychology (No.20/02.05.2023). The studies were conducted in accordance with the local legislation and institutional requirements. Written informed consent for participation in this study was provided by the participants / the participants’ legal guardians/next of kin.

## Author contributions

MM: Conceptualization, Formal analysis, Investigation, Methodology, Resources, Software, Validation, Visualization, Writing – original draft, Writing – review & editing. RR: Resources, Validation, Writing – original draft, Writing – review & editing. GC: Resources, Visualization, Writing – original draft, Writing – review & editing. GD: Resources, Visualization, Writing – original draft, Writing – review & editing.
